# Impact of primary dentition malocclusion on the oral health-related quality of life in preschoolers

**DOI:** 10.1186/s40510-021-00384-2

**Published:** 2021-11-22

**Authors:** Fernanda Marques Torres de Vasconcelos, Filipe Colombo Vitali, Marcos Ximenes, Loraine Fernandes Dias, Carla Pereira da Silva, Adriano Ferrati Borgatto, Michele Bolan, Mariane Cardoso

**Affiliations:** 1 Postgraduate Program in Dentistry, Department of Dentistry, Federal University of Santa Catarina, Florianopolis, Santa Catarina Brazil; 2 Department of Dentistry, University of Southern Santa Catarina, Palhoça, Santa Catarina Brazil; 3 Department of Informatics and Statistics, Federal University of Santa Catarina, Florianopolis,, Santa Catarina Brazil; 4 Department of Dentistry, Federal University of Santa Catarina, Florianopolis, Santa Catarina Brazil

**Keywords:** Malocclusion, Preschool, Primary dentition, Quality of life

## Abstract

**Background:**

Malocclusion is a condition frequently seen in primary dentition due to the interaction of environmental, genetic and behavioural factors. The occurrence of some types of malocclusions can have an impact on oral health-related quality of life in children. Hence, the present study aimed to verify the impact of primary dentition malocclusion on oral health-related quality of life in preschool children.

**Methods:**

A population-based cross-sectional study was conducted in Florianopolis, Brazil, with a representative sample of 1050 preschoolers aged between 2 and 5 years, randomly selected. Parents answered the Brazilian version of the Early Childhood Oral Health Impact Scale and also to a questionnaire on socio-economic indicators. Data obtained from the questionnaire were obtained by item response theory based on model of gradual response. The malocclusion assessed was: anterior open bite, increased overjet and posterior crossbite. Poisson regression model was employed for multivariate analysis (*P* < 0.05).

**Results:**

Malocclusion was observed in 36.7% of the children. Of these, 11.4% were anterior open bite, 67.2% were increased overjet, and 21.4% were posterior crossbite. Malocclusion's impact on oral health-related quality of life was 28.6%. In children aged 4–5 years, the prevalence of malocclusion’s impact on quality of life was 49.5% higher than in children aged 2–3 years. Statistical analysis showed that preschool children with malocclusion showed no significant impact on quality of life.

**Conclusions:**

The findings of the present study indicate that the occurrence of primary dentition malocclusion has no impact on the quality of life of children aged 2–5 years.

## Background

In recent years, subjective indicators have been developed to assess individuals' perception of their oral health, above and beyond a clinician’s opinion, such as measures of oral health-related quality of life (OHRQoL) [1–3]. The concept of OHRQoL is highly individual and involves the role of physical, psychological and social conditions on individuals’ well-being [2]. For public health purposes, oral health can be quantified at the macrolevel using the societal measures of oral conditions, which demonstrate that oral disease creates a substantial burden of illness [4]. Important gaps are observed in the evidence on the association between oral health conditions and OHRQoL, and such difficulty arises from the subjective assessment of patients in relation to aspects that can influence oral health [2]. To overcome this difficulty, multiple items questionnaires of OHRQoL have been developed to assess various criteria from the patient's point of view, expanding the research field [5].

Dental anomalies cause functional, occlusal and aesthetic problems that can result in oral health impairment [6]. Malocclusion is a condition frequently seen in primary dentition due to the interaction of environmental, genetic and behavioural factors [7, 8]. It is a condition that differs from the others because it is a change in the positions of the maxillary bones and/or teeth, rather than being a disease [9]. Therefore, the treatment is different from other conditions involving an orthodontic procedure to stabilize the occlusion [10]. It is known that the occurrence of some types of malocclusions frequently observed in children, such as anterior open bite, increased overjet and posterior crossbite, can produce functional and aesthetic effects that affect quality of life [1, 6, 11, 12].

Change that causes deviation from normality can stigmatize the person and often make it less socially acceptable [6, 13]. Evidence suggests that individuals with occlusal features with deviation from normality may attract unfavourable social responses, and such early life experiences may leave a permanent mark [13, 14]. Thus, the perception of young patients and their parents about malocclusion and its impact on daily life should not be neglected.

To assess subjective perceptions such as pain, aesthetics and function, indicators of OHRQoL are used to determine the impact of oral conditions [1]. The Early Childhood Oral Health Impact Scale (ECOHIS) is a questionnaire used to assess the impact of oral conditions on the quality of life of preschool children aged 2 to 5 years and their families in epidemiological research [3, 15]. It has been translated to Portuguese and validated for the use in Brazilian population [16, 17]. Therefore, the purpose of this study was to determine the impact of primary dentition malocclusion on the OHRQoL in preschool children and their families.

## Materials and methods

### Ethical considerations

This study received approval from the Human Research Ethics Committee (n. 343,658). Children’s caregivers read and signed a statement of informed consent prior to their participation.

### Study design

A cross-sectional study with a population-based sample was carried out to estimate the prevalence of malocclusion in primary teeth in children aged 2–5 years enrolled in public preschools of Florianopolis, Santa Catarina, Brazil, in 2018. According to the latest census (2015), the estimated population in the city is 485,838 people, 6349 children aged 2–5. The sample size calculation was based on a previous study [18] and considered 32.5% prevalence rate of malocclusion in preschool children. The G*Power 3 analysis (version 3.1, University Dusseldorf, Germany) was used. The standard error taken was 0.03 and the power (1-β error probability) 0.80. The required sample size was 937, and to balance for possible losses, 20% was added reaching 1124 pairs of children/parent. From the 72 public preschools in the city, 46 were randomly enrolled in the survey. A rating of the number of children aged 2 to 5 years enrolled in each preschool was performed and children were randomly selected following a system of the proportionality. From the 3 examiners, 2 were randomly selected for data collection in each preschool.

### Inclusion and exclusion criteria

To be included in the survey, children had to be aged 2–5 years old, regularly enrolled in preschool, present during the examination, with complete primary dentition and with parental consent. Furthermore, children with erupted permanent teeth, absence of the upper central incisors, previously orthodontically treated or uncooperative behaviour were excluded.

### Calibration of exams and pilot study

Three calibrated postgraduate dentists performed oral examinations (2 in each preschool), and a paedodontist, paediatric dentistry PhD, was considered the gold standard. The calibration exercise consisted of two steps. At first, the training exercise for malocclusion was done using images of different clinical situations, involved a discussion of the criteria established for diagnosing on two occasions with a 15-day interval. The second step was clinical, where twelve children were examined. The interval between assessments was 7–14 days. A pilot study was conducted at a day care centre with twenty-seven children, to test the methodology and understanding of the instruments. Children who participated in the pilot study were not in the main sample. Kappa coefficient was used to assess inter- and intra-examiner agreement for malocclusions diagnosis.

### Collection of non-clinical data

The Brazilian version of ECOHIS (B-ECOHIS) [16] was used to assess the children’s OHRQoL. Parents received the questionnaires at home through children school's diaries. B-ECOHIS considers the child’s entire life time’s experience of dental disease and treatment in parent’s responses. The B-ECOHIS questions assess six domains, four are on the child impact section: symptoms—one item; function—four items; psychological—two items; self-image/social interaction—two items; and two domains are on the family impact section: parent distress—two items and family function—two items. Response categories for the B-ECOHIS are coded: (0) never; (1) hardly ever; (2) occasionally; (3) often; (4) very often; and (5) don’t know. Questionnaires with more than three unanswered questions were excluded from the analysis.

To determine the socio-economic data of the family, parents/guardians answered a socio-economic questionnaire. In addition, a questionnaire with questions such as: gender, birth date and issues related to maternal breastfeeding and non-food sucking habits (pacifier and digital sucking) was applied.

### Clinical data collection

Oral examinations were performed in each selected school with children sitting in front of the examiner with the aid of a flashlight, clinical mirror, and when necessary, for improved visualization, teeth were cleaned and dried using sterile gauze. Examiners used personal protective equipment. A visual assessment of the children’s smile was also performed before the oral examination in a conversational way to establish whether aesthetics were compromised; examinations at a 50 cm distance evaluated the aesthetics effects observing colour changes of the anterior maxillary teeth crowns, as well as absence/fracture or dental caries.

The malocclusion assessed was: anterior open bite, increased overjet and posterior crossbite. They were evaluated with the teeth in maximum intercuspation. Anterior open bite: lack of a normal vertical superposition in any of the anterior incisors [19, 20]. For further analysis of the data, the dichotomization of the anterior open bite records was done in the absence or present, being considered present when ≥ 3 mm [19]. Increased overjet: the horizontal distance between the incisal edges of upper and lower central incisors greater than or equal to 3.1 mm [21], was classified as absent or present. Posterior crossbite was also classified as absent or present [20]. All data were recorded in a specific clinical file. The presence of at least one of these malocclusions classified the child as having malocclusion. The examiners also collected the following not clinical data: name, gender and age.

### Statistical analysis

Data analysis included a descriptive statistic for malocclusion variables anterior open bite, increased overjet and posterior crossbite, and non-clinical (such as B-ECOHIS data, socio-economic, maternal breastfeeding and non-nutritive sucking habits). Poisson regression was performed to verify the associations between quality of life with malocclusions, as well as determine associations of socio-economic data, maternal breastfeeding and non-nutritive sucking habits with as investigated malocclusions. Associations that had *P* < 0.20 were included in the adjusted regression. The database was made in Microsoft Excel 2013, and a statistical analysis was made using the Statistical Package for Social Sciences (SPSS) program. The level of significance was set at 5% (*P* < 0.05), and the confidence interval was 95%.

For the purpose of analysing the data obtained from the questionnaire was applied the mathematical model item response theory (IRT). This model sends a response to each item of the questionnaire (instrument) used and not use score from all the answers together. The advantage and the differential of this method are the concept of individualizing a sample by means of the latent trace, which is a characteristic of the individual as measured by each item of the instrument [22]. In applying the gradual response model from IRT to B-ECOHIS, each child received the score according to the latent trait [23]. These scores were set by the parents' response pattern of each child in B-ECOHIS. For a statistical analysis, the quality of life according to B-ECOHIS was dichotomized: "no impact" and "with impact".

## Results

A total of 1050 children were examined. The participation rate was 93.5%, and the main reasons for non-participation were lack of information and absence on the day of the examination. However, the socio-economic questionnaire with information about family income, parental education, prematurity and low birth weight had a low rate of return to researchers, which resulted in a lower response rate (less than 51.0%).

Table 1 presents an overview of the sample. The kappa coefficient reached inter- and intra-examiner values for malocclusion from 0.72 to 0.80. Of the 1050 children examined (mean age 3.8 ± 0.9 years), 48.7% were female (mean age was 3.8 ± 0.4 years), and 51.3% were male (mean age was 3.7 ± 0.9 years). The prevalence of investigated malocclusion was 36.7%. Of these, 80% had one type of malocclusion, 18.4% had two types, and 1.6% exhibit the three types of malocclusion. Among investigated malocclusion, 11.4% were anterior open bite, 67.2% were increased overjet, and 21.4% were posterior crossbite.

**Table 1:** Descriptive analysis of the data collected (*n* = 1050)

Variables	*n*	%
*Non-clinical data*		
Gender		
Female	511	48.7
Male	539	51.3
Age		
2 years	130	12.4
3 years	286	27.2
4 years	341	32.5
5 years	293	27.9
Quality of life		
No impact	738	70.3
With impact	312	29.7
*Clinical data*		
No investigated malocclusion	665	63.3
With investigated malocclusion	385	36.7

Table 2 presents the descriptive analysis of the items of B-ECOHIS, in which it is possible to observe that more than 70% of the subjects reporting “never” experiencing problems for all the questions.

**Table 2:** Descriptive analysis of questions related to the B-ECOHIS questionnaire (*n* = 1050)

Question	Never		Hardly ever		Occasionally		Often		Very often		Don’t know	
	*n*	%	*n*	%	*n*	%	*n*	%	*n*	%	*n*	%
*Child impact*												
Related pain	765	72.9	112	10.7	133	12.7	6	0.6	4	0.4	25	2.4
Had difficulty drinking hot or cold beverages	920	87.6	44	4.2	51	4.9	8	0.7	1	0.1	23	2.2
Had difficulty eating some foods	908	86.5	45	4.3	71	6.8	6	0.6	4	0.4	12	1.1
Had difficulty pronouncing words	887	84.5	28	2.7	51	4.9	15	1.4	7	0.7	60	5.7
Missed preschool, day care or school	939	89.4	45	4.3	57	5.4	4	0.4	1	0.1	3	0.3
Had trouble sleeping	942	89.7	35	3.3	43	4.1	7	0.7	4	0.4	14	1.3
Been irritable or frustrated	880	83.8	59	5.6	77	7.3	7	0.7	2	0.2	20	1.9
Avoided smiling or laughing	1010	96.2	13	1.2	13	1.2	3	0.3	3	0.3	8	0.7
Avoided talking	1005	95.7	19	1.8	12	1.1	1	0.1	1	0.1	9	0.9
*Family impact*												
Been upset	837	79.7	38	3.6	127	12.1	20	1.9	15	1.4	7	0.7
Felt guilty	811	77.2	39	3.7	116	11.0	25	2.4	12	1.1	8	0.7
Taken time off work	918	87.4	37	3.5	77	7.3	9	0.9	0	0.0	5	0.5
Financial impact	932	88.8	27	2.6	64	6.1	12	1.1	5	0.5	8	0.7

**Fig. 1:**
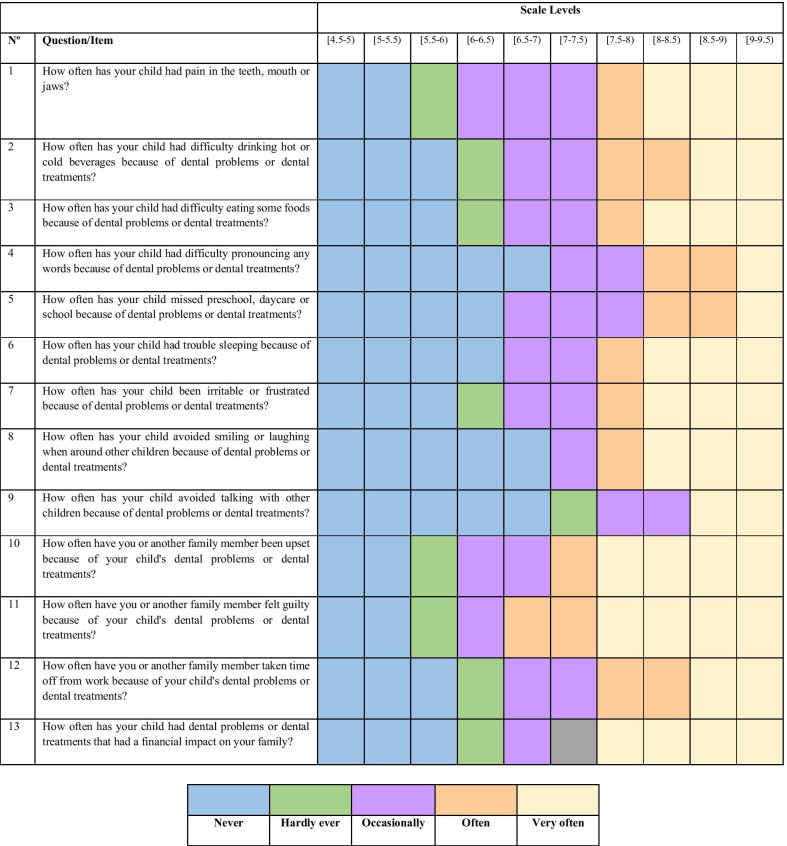
Placement of the items’ (answer) categories at the item response theory

Figure 1 shows the positioning of the categories of the items (answers) on the scale of IRT. From this, cut points were established taking into account the clinical representation of each item and the distributions of answers. The categories of items that represent the absence of impact of OHRQoL, represented by green, are mostly positioned at the start of the scale (left). Furthermore, the categories of items that represent the greatest impact on quality of life represented by parameter red are mostly positioned at the end of the scale (right). After analysing Fig. 1, it was possible to develop the scale divided into two levels: (1) No impact [4.5–5.5) 69%: at this level, the child never has issues related to the teeth and never need dental care; (2) With impact [5.5–9.5) 31%: at this level, the child hardly has trouble drinking hot or cold beverages; eating certain foods; gets angry; hardly a family member misses work; feels guilty and the child brings financially impact to the family income because of dental problems or dental care. Occasionally the child feels pain related to the teeth, mouth or jaw; occasionally a family is upset; and feels guilty because of dental problems or dental treatments of the child. The child very often presents problems related to the teeth and very often need dental care.

Table 3 presents the crude and adjusted Poisson regression analysis of the children grouped according to the number of malocclusions diagnosed in association with the OHRQoL. Only the children with one of the malocclusions analysed were included in the analysis. There was a significant association between the impact on the OHRQoL and age, showing that children aged 4 to 5 years have a prevalence of impact on OHRQoL of 49.5% higher than children aged 2 to 3 years. Statistical analysis showed that children with malocclusion showed no significant impact on quality of life.

**Table 3:** Univariate and multivariate Poisson regression model for independent variables and for impact on quality of life (*n* = 1050)

Variable	Impact on quality of life		Bivariate		Multivariate	
	Absent	Present	Unadjusted prevalence ratio		Adjusted prevalence ratio	
	n (%)	n (%)	*P*	(95% CI)	*P*	(95% CI)
*Gender*						
Female	352 (68.9)	159 (31.1)		1.00		
Male	386 (71.6)	153 (28.4)	0.542	0.937 (0.762–1.154)	–	–
*Age*						
2–3 years	317 (76.2)	99 (23.8)		1.00		
4–5 years	421 (66.4)	213 (33.6)	0.001	1.487 (1.187–1.863)	** < 0.001**	**1.495 (1.193–1.873)**
*Malocclusion*						
No	463 (69.6)	202 (30.4)		1.00		
Yes	275 (71.4)	110 (28.6)	0.473	0.923 (0.742–1.148)	–	–
*Anterior open bite*						
No	719 (70.8)	296 (29.2)		1.00		
Yes	19 (54.3)	16 (45.7)	0.132	1.454 (0.893–2.367)	0.096	1.514 (0.929–2.466)
*Increased overjet*						
No	589 (69.9)	254 (30.1)		1.00		
Yes	149 (72.0)	58 (28.0)	0.490	0.910 (0.695–1.190)	–	–
*Posterior crossbite*						
No	688 (69.9)	296 (30.1)		1.00		
Yes	50 (75.8)	16 (24.2)	0.461	0.841 (0.530–1.334)	–	–

A total of 534 (50.9%) children returned the questionnaire with socio-economic and suction habits information. As for socio-economic data, 19.1% had less than eight years of schooling and 62.7% had income less than or equal to three minimum wages.

## Discussion

This cross-sectional study was performed following the Strobe guidelines [24]. The main finding was that the prevalence of malocclusion was moderate with almost 4 in 10 children presenting some type of malocclusion, anterior open bite being the most frequent. However, it has no impact on the quality of life of the children neither they family.

The strength of the study was the adequate sample size, randomized sample and the high participation rate, which provides confidence to the results. As a limitation, only preschool children from public school were enrolled in the investigation, which means prudence is required in the generalizations of these findings. Moreover, relevant information regarding socio-economic status, parental education and sucking habits was not properly assessed since the response rate of socio-economic questionnaire was low.

In the present study, the items properly represented latent traits originated by responses to the questionnaire. Therefore, based on the data collected and analysed, it was possible to create an OHRQoL rating scale. IRT is a powerful tool that enables the construction of standardized scales from a set of items via mathematical models [23]. That is, the quality of the instrument is evaluated for each item that composes the questionnaire. The scale was developed with the scale intervals distributed between two levels (no impact, with impact) established by the researchers of this study. The decision for the cut-off was based on the distribution of the categories on the scale regions and clinical significance of the response of each category of items. The higher the scale interval of the index, the greater the impairment of the oral health of children. Approximately 69% of the children in our sample fell within the initial part of the scale, indicating that these children were not affected on their quality of life. Similar rates (65–70%) were noted by other studies using data from the same instrument [25, 26].

This investigation observed that regardless of the type or number of malocclusion, it does not practice a negative impact on an OHRQoL. Similar results were reported in other studies on preschool children [1, 17, 26]. Despite the similarity between the results, there are significant methodological differences. In addition to the age-related differences of the children included in these studies, we observed differences in the assessment of malocclusion. In some investigations, the association between malocclusion and negative impact on quality of life was made considering the presence or absence of at least one type of malocclusion, whereas in other studies, the impact of each type of malocclusion on OHRQoL was assessed [27, 28]. Differences were also observed in the types of malocclusions assessed [15]. The lack of association may be attributed to malocclusions having mainly aesthetic implications, not prioritized by preschool children. Thus, this change does not cause a negative oral impact on young children, but is more relevant among older children, whose maturity disposes them to evaluate aesthetic aspects [12, 26].

Dental pain, difficulty of eating and difficulty pronouncing words were the most frequently answered items in the B-ECOHIS. This result is consistent with other studies on preschool children [15, 26, 28]. The child’s limitation in performing these activities is more easily perceived by parents of preschool children than the child’s aesthetic aspects. Also, at this age, children are not mature enough to compare their self-image to others, and thus, they complain to a lesser degree. Consequently, complaints about pain and difficulty eating are more common among children, thus explaining the increased frequency of these aspects in the B-ECOHIS.

This study found that among the occlusal relationships evaluated, even in these cases where aesthetic impairments are present, such as in cases of anterior open bite or increased overjet, it does not seem to be a problem for children or their parents. A similar study with children aged 11–14 years showed that the most significant impact of malocclusion on OHRQoL is in the psychosocial field, affecting emotional well-being and social domains [11]. The results found with preschool children were different. The difference in results may be related to malocclusions evaluated in this study, as they are often associated with non-nutritive sucking habits, such as the sucking of a finger or pacifier, and also the prolonged use of a bottle-feeding. Therefore, aesthetics changes produced by maintenance of these habits are usually considered as acceptable [1].

There is increasing interest in assessing the impact of malocclusion on a child's psychosocial well-being. Childhood experiences can play a significant role in the following years, where a negative dental appearance can be embarrassing for other children [12]. An important clinical implication of the present study is that strategies to promote oral health decrease the prevalence of malocclusion. It is important to evaluate school-age children with mixed and primary dentition, as an early diagnosis can contribute to preventive or interceptive orthodontics, taking advantage of the child's growth potential. The present study corroborates with data in the literature that reports that despite moderate prevalence, malocclusions have little impact on the quality of life of children 2 to 5 years of age. The findings can be cautiously generalized to populations with cultural and demographic characteristics similar to populations living in southern Brazil, which is composed mainly of individuals in different socio-economic standard in a developing country.

## Conclusions

The results of this present study revealed that OHRQoL scores among preschoolers aged 2 to 5 years are not affected by primary dentition malocclusion.

## Data Availability

Data are available on justified request to the authors.

## Abbreviations

B-ECOHIS: Brazilian version of Early Childhood Oral Health Impact Scale
ECOHIS: Early Childhood Oral Health Impact Scale
IRT: Item response theory
OHRQoL: Oral Health-related Quality of Life
